# Digenean trematodes infecting the frigate tuna *Auxis thazard* (Scombriformes, Scombridae) off the Rio de Janeiro coast, Brazil, including molecular data[Fn FN1]

**DOI:** 10.1051/parasite/2022044

**Published:** 2022-10-07

**Authors:** Camila Pantoja, Bruno Telles, Fabiano Paschoal, José Luis Luque, Olena Kudlai

**Affiliations:** 1 Institute of Ecology, Nature Research Centre Akademijos 2 08412 Vilnius Lithuania; 2 Departamento de Parasitologia Animal, Universidade Federal Rural do Rio de Janeiro BR 465 km 7 23890-000 Seropédica Rio de Janeiro Brazil; 3 Programa de Pós-Graduação em Biodiversidade e Conservação, Departamento de Oceanografia e Limnologia, Uiversidade Federal do Maranhão Av. dos Portugueses 1966 65080-805 São Luís Maranhão Brazil

**Keywords:** Bucephalidae, Didymozoidae, Fellodistomidae, Hemiuridae, Mitochondrial and nuclear DNA, Southwestern Atlantic

## Abstract

Although some parasitological efforts have focused on the frigate tuna *Auxis thazard* (Lacepède) (Scombriformes, Scombridae) in Brazil, its digenean fauna remains poorly known. Combining morphological and molecular methods, we investigated the diversity of digenean trematodes of *A*. *thazard* collected from the coastal waters off the state of Rio de Janeiro, Brazil in 2021. Six species belonging to four families were recorded: the bucephalid *Rhipidocotyle* cf. *angusticolle* Chandler, 1941, the didymozoid *Didymocystis* sp. 6 *sensu* Louvard et al. (2022), the fellodistomid *Tergestia* sp., and three hemiurids, *Dinurus euthynni* Yamaguti, 1934, *Lecithochirium floridense* (Manter, 1934), and *L*. *synodi* Manter, 1931. The current study brings the total number of digenean trematode species parasitising *A*. *thazard* in Brazil up to eight, with hemiuroid trematodes being most diverse. *Auxis thazard* is a new host record for *L. floridense*, *L. synodi* and potentially for *R. angusticolle*. The geographic distribution of several species found in our study appeared to be wider than previously known. Our study is the first to apply a DNA-based approach to digenean diversity in marine fishes in Brazil and we believe that both morphological descriptions and molecular sequence data provided in our study will aid future research assessing the diversity of digenean trematodes of *A*. *thazard* and other marine fishes in Brazil.

## Abstract (fr)

Bien que certains efforts parasitologiques aient porté sur l’auxide *Auxis thazard* (Lacepède) (Scombriformes : Scombridae) au Brésil, la faune de ses digènes reste mal connue. En combinant des méthodes morphologiques et moléculaires, nous avons étudié la diversité des trématodes digènes d’*A. thazard* collectés dans les eaux côtières au large de l’État de Rio de Janeiro, Brésil en 2021. Six espèces appartenant à quatre familles ont été répertoriées : le Bucephalidae *Rhipidocotyle* cf. *angusticolle* Chandler, 1941, le Didymozoidae *Didymocystis* sp. 6 *sensu* Louvard et al. (2022), le Fellodistomidae *Tergestia* sp., et trois Hemiuridae, *Dinurus euthynni* Yamaguti, 1934, *Lecithochirium floridense* (Manter, 1934) et *L. synodi* Manter, 1931. L’étude actuelle porte le nombre total d’espèces de trématodes digènes parasitant *A. thazard* au Brésil à huit, les Hemiuroidea étant les plus diversifiés. *Auxis thazard* est un nouveau signalement d’hôte pour *L. floridense*, *L. synodi* et potentiellement *R. angusticolle*. La répartition géographique de plusieurs espèces trouvées dans notre étude semble être plus large que ce que nous savions auparavant. Notre étude est la première à appliquer une approche basée sur l’ADN à la diversité des digènes chez les poissons marins au Brésil et nous pensons que les descriptions morphologiques et les données de séquence moléculaire fournies dans notre étude aideront les recherches futures évaluant la diversité des trématodes digènes d’*A. thazard* et d’autres poissons marins du Brésil.

## Introduction

The Brazilian marine fish fauna is exceptionally diverse and encompasses over 1200 species which comprise nearly 7% of the world’s marine fish species [30, 77]. These fishes certainly host a rich diversity of parasites. However, literature suggests that our knowledge on this diversity is fragmentary and incomplete due to differential study efforts and uneven geographical coverage; most studies have been conducted off the southern coast in contrast to the north–eastern Atlantic coast [9]. Although helminth parasites have only been reported from a small portion of fishes (less than 20%) in Brazilian marine ecosystems, existing records suggest that digenean trematodes are one of the most diverse groups of parasites [41, 52]. To date, there are reports of 184 species from 28 families of digenean trematodes, with the families Didymozoidae Poche, 1907, Hemiuridae Looss, 1899, and Opecoelidae Osaki, 1925 being the most diverse in the region [6, 21, 38, 41]. The most investigated fish family is the Carangidae followed by the Sciaenidae and Scombridae. All previous records are based on morphology and to the best of our knowledge, no attempts applying a molecular methodology have been made to address the taxonomic diversity of marine digenean trematodes in Brazil. Thus, it can be expected that many unknown digenean trematode species await their discovery, and many known species are yet to be genetically characterised.

The present study was carried out within a short-term project aiming to discover and morphologically and molecularly characterise the diversity of trematode species of marine fishes off the Brazilian Atlantic coastline. The current paper reports on the findings of a survey on digenean trematodes parasitizing the scombrid *Auxis thazard* (Lacépède) collected from the coastal waters off the state of Rio de Janeiro. Commonly known as frigate tuna, this pelagic fish is an important component of marine food webs and has high economic importance [25, 29, 30]. *Auxis thazard* is a widespread species found in the Atlantic, Indian, and Pacific (Western and Central) oceans [30] and, due to its ubiquity, numerous studies investigating trematodes of this fish have been conducted around the world (Table 1). These studies recorded digenean trematodes from at least six families, of which the Didymozoidae is by far the most speciose. Most records of the trematode fauna of *A*. *thazard* derive from the Indo-west Pacific region (the South China Sea and Hawaii). The digenean trematode fauna of *A*. *thazard* in Brazil is yet to be comprehensively assessed; however, two species have been reported: the bucephalid *Rhipidocotyle pentagonum* (Ozaki, 1924) [24] and the didymozoid *Melanocystis kawakawa* Yamaguti, 1970 [38]. In Brazil, *A. thazard* is also known as a host for one species of acanthocephalans [*Rhadinorhynchus pristis* (Rudolphi, 1802)], six species of monogeneans [*Allopseudaxine macrova* (Unnithan, 1957), *Capsala magronum* (=*Caballerocotyle lenti*) (Ishii, 1936), *Churavera triangula* (Mamaev, 1967), *Hexostoma thynni* (=*Hexostoma auxisi*) (Delaroche, 1811), *H. keokeo* Yamaguti, 1968 and *Sibitrema* sp.] and three species of nematodes [*Anisakis typica* Diesing, 1860, *An*. *physetesis* (Baylis, 1923), *Oncophora melanocephala* (Rudolphi, 1819)] [21, 36, 39, 51, 61, 64, 66, 72]. Our study further explores the diversity of trematodes and suggests that *A*. *thazard* off the Brazilian Atlantic coastline is infected by at least eight species from four families. Details of morphology, novel DNA sequence data, and host and geographical records are presented.

**Table 1:** Summary data on digenean trematodes found in *Auxis thazard* (Lacepède).

Family	Species	Locality	Reference
Bucephalidae	*Prosorhynchoides gracilescens*	Adriatic Sea	[82]
Bucephalidae	*Prosorhynchus* sp.	South China Sea	[56]
Bucephalidae	*Rhipidocotyle capitata*	Hawaii, USA	[94]
Bucephalidae	*Rhipidocotyle nagatyi*	Mauritania	[86]
Bucephalidae	*Rhipidocotyle pentagonum*	Rio de Janeiro, Brazil; Bay of Bengal, India; South China Sea	[24, 55, 56]
Didymozoidae	*Annulocystis auxis*	Hawaii, USA; Moreton Bay, Australia	[94, 49]
Didymozoidae	*Annulocystis* sp. 1	Bali, Indonesia	[49]
Didymozoidae	*Annulocystis* sp. 2	Moreton Bay, Australia	[49]
Didymozoidae	*Colocyntotrema auxis*	Taizi, Japan; Moreton Bay, Australia	[93,49]
Didymozoidae	*Didymocystis dissimilis*	South China Sea	[56]
Didymozoidae	*Didymocystis exigua*	Bay of Bengal	[67]
Didymozoidae	*Didymocystis wedli*	Bay of Bengal; Muara Baru, Indonesia	[53, 87]
Didymozoidae	*Didymocystis* sp. 6	Moreton Bay, Australia	[49]
Didymozoidae	*Didymosphaera mirabilis*	North Vietnam Gulf, Vietnam	[56]
Didymozoidae	*Didymozoon auxis*	Taizi, Japan	[93]
Didymozoidae	Didymozoidae gen. sp.	Muara Baru, Indonesia	[87]
Didymozoidae	Didymozoidae gen. sp.	South China Sea	[56]
Didymozoidae	Didymozoidae gen. sp. larvae	South China Sea	[56]
Didymozoidae	Didymozoidae gen. sp.	Moreton Bay, Australia	[49]
Didymozoidae	Didymozoidae gen. sp.	Moreton Bay, Australia	[49]
Didymozoidae	*Koellikerioides orientalis*	Muara Baru, Indonesia	[87]
Didymozoidae	*Lobatozoum multisacculatum*	South China Sea	[56]
Didymozoidae	*Melanocystis kawakawa*	Rio de Janeiro, Brazil	[38]
Didymozoidae	*Metanematobothrium bivitellatum*	South China Sea	[56]
Didymozoidae	Nematobothriinae sp. 1	Moreton Bay, Australia	[49]
Didymozoidae	*Opepherotrema planum*	Taizi, Japan	[93]
Didymozoidae	*Phacelotrema claviforme*	Taizi, Japan	[93]
Didymozoidae	*Pseudocolocyntotrema yaito*	Hawaii, USA	[94]
Didymozoidae	*Sicuotrema auxis*	Hawaii, USA	[95]
Fellodistomidae	*Tergestia laticollis*	Tortugas, Florida, USA	[45, 59]
Gorgoderidae	*Phyllodistomum lancea*	South China Sea	[56]
Hemiuridae	*Brachyphallus parvus*	Gulf of Mexico	[50]
Hemiuridae	*Dinurus euthynni*	South China Sea	[56]
Hemiuridae	Dinurinae gen. sp.	Gulf of Mexico	[50]
Hemiuridae	*Ectenurus* sp.	Tortugas, Florida, USA	[45]
Hemiuridae	*Lecithochirium imocavum*	South China Sea	[56]
Hemiuridae	*Lecithochirium keokeo*	Hawaii, USA	[94]
Hemiuridae	*Lecithochirium magnaporum*	Hawaii, USA	[94]
Hemiuridae	*Lecithochirium* sp.	Muara Baru, Indonesia	[87]
Hemiuridae	*Plerurus digitatus*	South China Sea; Hawaii, USA	[56, 94]
Hirudinellidae	*Hirudinella* sp.	Tsushima Islands, Japan	[50]

## Material and methods

### Ethics statement

All applicable institutional, national and international guidelines for the ethical handling of animals were followed. According to Brazilian laws, species registration for scientific research purposes was carried out at SisGen (Number AFB3925).

### Sampling and morphological evaluation

Three specimens of *A. thazard* (total length 30–40 cm) were obtained from local fisherman in Cabo Frio coastal zone (22°52′46″ S, 42°01′07″ W), State of Rio de Janeiro, Brazil in January 2021. The fish host nomenclature follows Froese and Pauly [30]. Fish were dissected fresh and examined for the presence of helminths. Trematodes were rinsed in 0.9% saline and fixed in 4% hot formalin (paragenophores according to Pleijel et al. [71]) and in molecular grade 96% ethanol. Cysts containing trematodes were removed from the stomach tissue of fish using needles. Specimens selected for molecular genetic study, i.e., hologenophores were processed as described in Faltýnková et al. [23]. Hologenophores and formalin-fixed specimens transferred to 70% ethanol were stained with Mayer’s hydrochloric carmine solution, dehydrated in an ascending ethanol series, cleared with eugenol (clove oil), mounted in Canada balsam and thereafter, used for morphological study. Drawings were made using a drawing tube attached to an Olympus BX 51 microscope. Measurements were taken using QuickPHOTO CAMERA 2.3 image analysis software adapted to an Olympus BX51 and are given in micrometres unless otherwise stated. The voucher specimens were deposited in the Helminthological Collection of the Oswaldo Cruz Institute (CHIOC).

### Molecular genetic evaluation

The methodology used for extraction of genomic DNA and generation of 28S rDNA and ITS2 sequences was identical to that described in Faltýnková et al. [23]. Two partial fragments of the *cox*1 gene were amplified using the primers JB3 (forward; 5′–TTT TTT GGG CAT CCT GAG GTT TAT–3′) [5] and CO1-R trema (reverse; 5′–CAA CAA ATC ATG ATG CAA AAG G–3′) [40], and Dig_cox1Fa (forward; 5′–ATG ATW TTY TTY TTY YTD ATG CC–3′) and Dig_cox1R (reverse; 5′–TCN GGR TGH CCR AAR AAY CAA AA–3′) [91] by PCR following the protocol published by Miura et al. [63] and Wee et al. [91], respectively. The amplified products were purified with Exo-SAP-IT KitTM Express Reagent (Thermo Fisher Scientific Baltics UAB, Vilnius, Lithuania), following the manufacturer’s instructions and sequenced using the Big Dye Terminator V3.1 Cycle Sequencing kit and ABI 3730 (XL) DNA Analyzer capillary sequencing robot (Applied Biosystems, Foster City, CA, USA). Sequencing was performed using the same primers as for PCR reactions and two additional primers, 300F and ECD2 [46] were used for 28S rDNA. Geneious v. 11 (Biomatters, Auckland, New Zealand) was used to assemble sequences. Novel sequences were deposited in GenBank with accession numbers OP418194– OP418196; OP424997– OP424998; OP458330– OP458341.

The Basic Local Alignment Search Tool (BLAST) (http://www.ncbi.nlm.nih.gov/blast) was used to compare sequences obtained in the present study to those available in GenBank. Four alignments including novel and previously published sequences were built using ClustalW implemented in Geneious v. 11. All four alignments were used for comparative sequence analysis (*p*-distance and nucleotide (nt) difference) and Alignment 4 was further used for Bayesian inference (BI) and maximum likelihood (ML) phylogenetic analyses. Alignment 1 (1220 nt) included 28S rDNA sequences of nine *Rhipidocotyle* spp.; one sequence generated in the present study. Alignment 2 (598 nt) included six ITS2 sequences of *Rhipidocotyle* spp.; one sequence generated in the present study. Alignment 3 (978 nt) included 12 28S rDNA sequences of the subfamily Didymozoinae; two sequences generated in the present study. Alignment 4 (1119 nt) included 23 28S rDNA sequences of the family Hemiuridae; four sequences generated in the present study. Distance matrices for the alignments were calculated in MEGA ver. X [43].

To assess phylogenetic relationships for Alignment 4, we used BI and ML analyses. Sequence of *Isoparorchis eurytremus* (Kobayashi, 1915) (MH628315), a parasite of *Silurus asotus* Linnaeus from Japan was used as the outgroup based on the results of the phylogenetic analyses of the superfamily Hemiuroidea published by Louvard et al. [49]. Prior to analyses, the best-fitting model was estimated with jModelTest 2.1.2 [20]. This was the general time-reversible model incorporating invariant sites and gamma distributed among-site rate variations (GTR+I+G). BI analysis was conducted using MrBayes software (ver. 3.2.3) [79] and run on the CIPRES portal [62]. Markov chain Monte Carlo (MCMC) chains were run for 10,000,000 generations, log-likelihood scores were plotted and only the final 75% of trees were used to produce the consensus trees. The results were submitted in Tracer ver. 1.6 [75] to evaluate proper sampling and to identify the “burn-in” period. ML analysis was conducted using PhyML version 3.0 [34] run on the ATGC bioinformatics platform (http://www.atgc-montpellier.fr/). Nodal support was estimated by performing 100 bootstrap pseudoreplicates. For the trees visualisation, FigTree ver. 1.4 software [74] was used.

To avoid ambiguity for some generic names, the following abbreviations were used: A., *Auxis*; Al., *Aluterus*; An., *Anisakis*; D., *Dinurus*; Di., *Didymocystis*; H., *Hexostoma*; He., *Hemiurus*; P., *Pterois*; Pa., *Paralichthys*; R., *Rhipidocotyle*; Rh., *Rhomboplites*; S., *Syacium*; Sy., *Synodus*; T., *Tergestia*; Th., *Thunnus*.

## Results

Morphological and molecular evaluation of collected specimens of digenean trematodes revealed the presence of six species from four families. One species belongs to the family Bucephalidae, one to the family Didymozoidae, one to the family Fellodistomidae and three to the family Hemiuridae. No larval stages of trematodes were recorded in examined fish. A total of 17 novel sequences were generated for five out of six species of trematodes: 28S rDNA (*n* = 7), ITS2 (*n* = 5) and *cox*1 (*n* = 5). Sequences of *Tergestia* sp. were not generated.

**Bucephalidae Poche, 1907**

***Rhipidocotyle* Diesing, 1858**

### *Rhipidocotyle* cf. *angusticolle* Chandler, 1941

*Site of infection*: stomach.

*Infection rates*: 1 out of 3; 1 specimen per fish.

*Representative DNA sequences*: OP458334 (28S); OP458341 (ITS2).

*Voucher material*: 1 voucher specimen (hologenophore) CHIOC–39762.

*Remarks*: Only one specimen of this species was collected. The specimen corresponds well to the generic diagnosis of the genus *Rhipidocotyle* Diesing, 1858 provided by Overstreet and Curran [69] in possessing rhynchus consisting of a simple sucker with muscular hood containing five large fleshy lobes, mouth near mid-body, vitellarium in two fields, anterior to ovary, and oblique testes. Morphology of our single specimen, although incomplete and bent, corresponds to the original description of *R*. *angusticolle* by Chandler [14], and later updated redescription based on the type and newly collected material by Corkum [17] by rhynchus as a simple sucker with five fleshy lobes and vitelline follicles arranged in symmetrical lateral fields (about 15 follicles each side). However, it differs from the original description by smaller eggs [15–18 × 9–13 (*n* = 20) *vs* 21–22 × 14–16].

*Rhipidocotyle angusticolle* is an intestinal parasite of scombrid fishes from the western Atlantic Ocean. The species was reported in *Sarda sarda* (Bloch) from Gulf of Mexico [14], and in *Euthynnus alletteratus* (Rafinesque) and *Scomberomorus cavalla* (Cuvier) off Grand Isle, Louisiana, USA [17, 68]. Shalaby and Hassanine [83] recorded *R*. *angusticolle* in the serranid *Epinephelus fasciatus* (Forsskål) in the Red Sea. However, the specimens were described possessing rhynchus without fleshy lobes and according to Bartoli and Bray [3] the species may not have been correctly identified. In Brazil, *R*. *angusticolle* was previously reported from *Scomber colias* Gmelin by Fabio [22]. *Auxis thazard* is a new host record for this species.

*Molecular results*: Comparative sequence analysis of Alignment 1 demonstrated that the sequence of the species found in our study (OP458334) exhibited the lowest divergence with the sequence of *R*. *angusticolle* (KT273383) obtained from *E. alletteratus* collected off Grand Isle, Louisiana, USA, i.e., 0.5% (6 nt). The interspecific divergence between *Rhipidocotyle* spp. in Alignment 1 ranged from 4.6 to 11.6% (51–127 nt) (Table 2). Comparative sequence analysis of Alignment 2 showed similar results. The difference between ITS2 sequences of our specimen (OP458341) and *R*. *angusticolle* (KT273383) was 1% (5 nt). This is rather low compared to the interspecific divergence in this dataset which ranged from 7 to 28% (35–140 nt) (Table 2). Intraspecific genetic variation in 28S and ITS2 data has been observed in previous studies of bucephalids. Cutmore et al. [19] reported intraspecific variation of 3 nt and 4 nt in the 28S and ITS2 datasets, respectively for specimens of *Dollfustrema durum* Nolan, Curran, Miller, Cutmore, Cantacessi & Cribb, 2015 collected from *Gymnothorax javanicus* (Bleeker) and *G*. *pseudothyrsoideus* (Bleeker) in Moreton Bay and from Great Barrier Reef. Corner et al. [18] reported intraspecific variation of 3 nt in the ITS2 dataset for *Aenigmatrema undecimtentaculatum* Corner, Cribb & Cutmore, 2020 (Bucephalidae) from *Sphyraena obtusata* Cuvier in Moreton Bay, Australia. Although the genetic divergence between sequences of our specimen and *R. angusticolle* is slightly higher, there have been similar results obtained by Bray et al. [8] who reported intraspecific variation of 5 nt in the 28S and 5 nt in the ITS2 datasets for a species of lepocreadiid, *Preptetos prudhoei* Bray, Cutmore & Cribb, 2021 from acanthurid fishes which was associated with geographical distribution (Heron Island *vs* French Polynesia). Considering the low level of sequence divergence between our specimen and *R*. *angusticolle* in relation to other congeners and similarities in morphology, we provisionally consider them as conspecific.

**Table 2:** Nucleotide comparison of the partial 28S rDNA sequences and ITS2 complete sequences of *Rhipidocotyle* spp. based on 1220 nt and 598 nt long alignments, respectively. P-distance (%) is given below diagonal and the number of variable nucleotides above diagonal.

	28S rDNA sequences	1	2		3		4	5		6		7	8		9
1	OP458334 *Rhipidocotyle* cf. *angusticolle*		6		78		82	98		108		106	109		114
2	KT273383 *Rhipidocotyle angusticolle*	0.5			77		86	99		109		107	110		115
3	AY222225 *Rhipidocotyle galeata*	6.5	6.5				85	88		101		98	103		108
4	KT273390 *Rhipidocotyle lepisostei*	6.9	7.2		7.1			111		127		112	124		126
5	KF184355 *Rhipidocotyle campanula*	8.3	8.4		7.4		9.3			81		45	82		69
6	MK648267 *Rhipidocotyle* sp.	9.9	10		9.2		11.6	7.4				83	51		83
7	KF184361 *Rhipidocotyle fennica*	9.0	9.1		8.3		9.4	3.8		7.6			78		71
8	KT273394 *Rhipidocotyle transversalis*	9.2	9.3		8.7		10.4	6.9		4.6		6.6			80
9	KT273384 *Rhipidocotyle tridecapapillata*	9.6	9.7		9.1		10.6	5.8		7.5		5.9	6.7		

	**ITS2 sequences**	1		2		3			4		5			6	

1	OP458341 *Rhipidocotyle* cf. *angusticolle*			5		106			115		122			125	
2	KT273383 *Rhipidocotyle angusticolle*	1.0				104			117		123			125	
3	KT273390 *Rhipidocotyle lepisostei*	20.6		20.2					140		133			131	
4	KF184365 *Rhipidocotyle fennica*	24.3		24.7		28.0					35			91	
5	KF184358 *Rhipidocotyle campanula*	25.5		25.7		26.2			7.0					94	
6	KT273394 *Rhipidocotyle transversalis*	27.2		27.2		27.0			19.1		19.5				

**Fellodistomidae Nicoll, 1909**

***Tergestia* Stossich, 1899**

### *Tergestia* sp.

*Site of infection*: stomach.

*Infection rates*: 1 out of 3; 2 specimens per fish.

*Voucher material*: 2 voucher specimens CHIOC–39763 a–b.

*Remarks*: Specimens found in the present study agree well with the generic diagnosis of *Tergestia* Stossich, 1899 provided by Bray [11] in having oral sucker papillate, muscular flanges on forebody at level of the pharynx, intestinal bifurcation postero-dorsal to the ventral sucker and uterus extending into the post-testicular region. Only two specimens of this species were collected, and their quality restricts observation of the key morphological features designated by Wee et al. [91] used for species differentiation and identification. The DNA sequences of this material were not generated. Therefore, we provide identification of the species to the genus level.

Currently 16 species are recognized within *Tergestia* [91]. In Brazil, three species have been reported to date: *T*. *laticollis* (Rudolphi, 1819) from the scombrid *Thunnus albacares* (Bonnaterre) [24], *T*. *pauca* Texeira de Freitas & Kohn, 1965 from carangids *Selene setapinnis* (Mitchill) and *Scomberoides* sp. [28, 89], and *T*. *selenei* Amato, 1983 from carangids *Caranx hippos* (L.) and *S*. *setapinnis* [2, 16]. Wallet and Kohn [89] considered *T*. *selenei* as a synonym of *T*. *pauca*; however, according to a recent study of Wee et al. [91] this species is valid. *Tergestia laticollis* is the only species of the genus previously reported from *A*. *thazard* in Tortugas, Florida, USA by Linton [45] and Manter [59] (Table 1).

**Didymozoidae Monticelli, 1888**

***Didymocystis* Ariola, 1902**

### *Didymocystis* sp. 6 *sensu* Louvard et al. (2022)

*Site of infection:* encysted in stomach tissue.

*Infection rates*: 1 out of 3; 9 specimens in total.

*Representative DNA sequences*: OP458335, OP458336 (28S); OP418196 (*cox*1).

*Voucher material*: 5 voucher specimens (4 in ethanol; 1 mounted on slide) CHIOC–39372, CHIOC–39758.

*Remarks*: Specimens of *Didymocystis* sp. 6 *sensu* Louvard et al. [49] were found in capsules in stomach tissue of one fish. Our identification of the species was confirmed based on comparative sequence analysis (see below). The worms were damaged in an attempt to remove them from the capsules, and therefore we do not provide morphological description and identification to the species level using morphological criteria. *Didymocystis* sp. 6 *sensu* Louvard et al. [49] is a parasite of the stomach of *A*. *thazard* recently reported from Moreton Bay, Queensland, Australia. This is the first record of this species off the Brazilian coast, southwestern Atlantic Ocean.

Digenean trematodes of the family Didymozoidae are common parasites of scombrid fishes [49, 54, 73]. This family is the most species rich in *A*. *thazard* with 24 nominal species reported (Table 1). In Brazil, the family Didymozoidae is represented by 34 species with eight species from the genus *Didymocystis*; all recorded from scombrids [21, 38]. *Melanocystis kawakawa* is the only didymozoid previously reported in *A*. *thazard* in Brazil [38].

*Molecular results*: Two 28S sequences of *Didymocystis* sp. 6 *sensu* Louvard et al. [49] generated in our study were identical. Comparative sequence analysis of Alignment 3 demonstrated that these sequences exhibited the lowest divergence with the sequence of *Didymocystis* sp. 6 *sensu* Louvard et al. [49]. The intraspecific divergence between three isolates was 0.2% (3 nt). This is rather low compared to the interspecific divergence in this dataset which ranged from 7.4 to 8.5% (75–88 nt) (Table 3). Considering the low level of sequence divergence between our specimens and *Didymocystis* sp. 6 *sensu* Louvard et al. [49], we consider them conspecific. Comparative sequence analysis between the *cox*1 sequence of *Didymocystis* sp. 6 generated in our study and sequences of *Didymocystis* spp. provided by Louvard et al. [49] demonstrated the lowest sequence divergence with *Didymocystis* sp. 5 (31.6%, 150 nt) and the highest sequence divergence with *Didymocystis* sp. 3 (34.5%, 164 nt).

**Table 3:** Nucleotide comparison of the partial 28S rDNA sequences of the Didymozoinae based on 978 nt long alignment. *P*-distance (%) is given below diagonal and the number of variable nucleotides above diagonal.

		1	2	3	4	5	6	7	8	9	10	11	12
1	OP458335 *Didymocystis* sp. 6		0	3	83	83	80	80	75	76	83	86	87
2	OP458336 *Didymocystis* sp. 6	0.0		3	83	83	80	80	75	77	83	86	87
3	OL336008 *Didymocystis* sp. 6	0.2	0.2		84	84	81	81	76	77	84	87	88
4	OL336002 *Didymocystis* sp. 1	8.2	8.2	8.2		7	48	54	49	50	7	82	82
5	OL336003 *Didymocystis* sp. 2	8.2	8.2	8.2	0.7		48	54	51	52	0	81	81
6	OL336004 *Didymocystis* sp. 3	7.7	7.7	7.7	4.7	4.7		29	26	27	48	69	67
7	OL336005 *Didymocystis* sp. 4	7.9	7.9	7.9	5.3	5.3	2.6		28	29	54	74	72
8	OL336006 *Didymocystis* sp. 5	7.4	7.4	7.4	5.0	5.2	2.5	2.7		1	51	67	65
9	OL336007 *Didymocystis* sp. 5	7.4	7.4	7.4	5.0	5.2	2.5	2.7	0.0		52	68	66
10	OL336009 *Didymocystis* sp. 7	8.2	8.2	8.2	0.7	0.0	4.7	5.3	5.2	5.2		81	81
11	KU341979 *Didymocystis scomberomori*	8.4	8.4	8.4	8.1	8.0	6.6	7.1	6.6	6.6	8.0		5
12	KU341980 *Didymocystis* sp.	8.5	8.5	8.5	8.1	8.0	6.4	6.9	6.4	6.4	8.0	0.5	

**Hemiuridae Looss, 1899**

**Dinurinae Looss, 1907**

***Dinurus* Looss, 1907**

### *Dinurus euthynni* Yamaguti, 1934

*Site of infection*: stomach.

*Infection rates*: 1 out of 3; 12 specimens in total.

*Representative DNA sequences*: OP458333 (28S); OP458340 (ITS2).

*Voucher material*: 9 voucher specimens CHIOC–39759 a–i.

**Figure 1:**
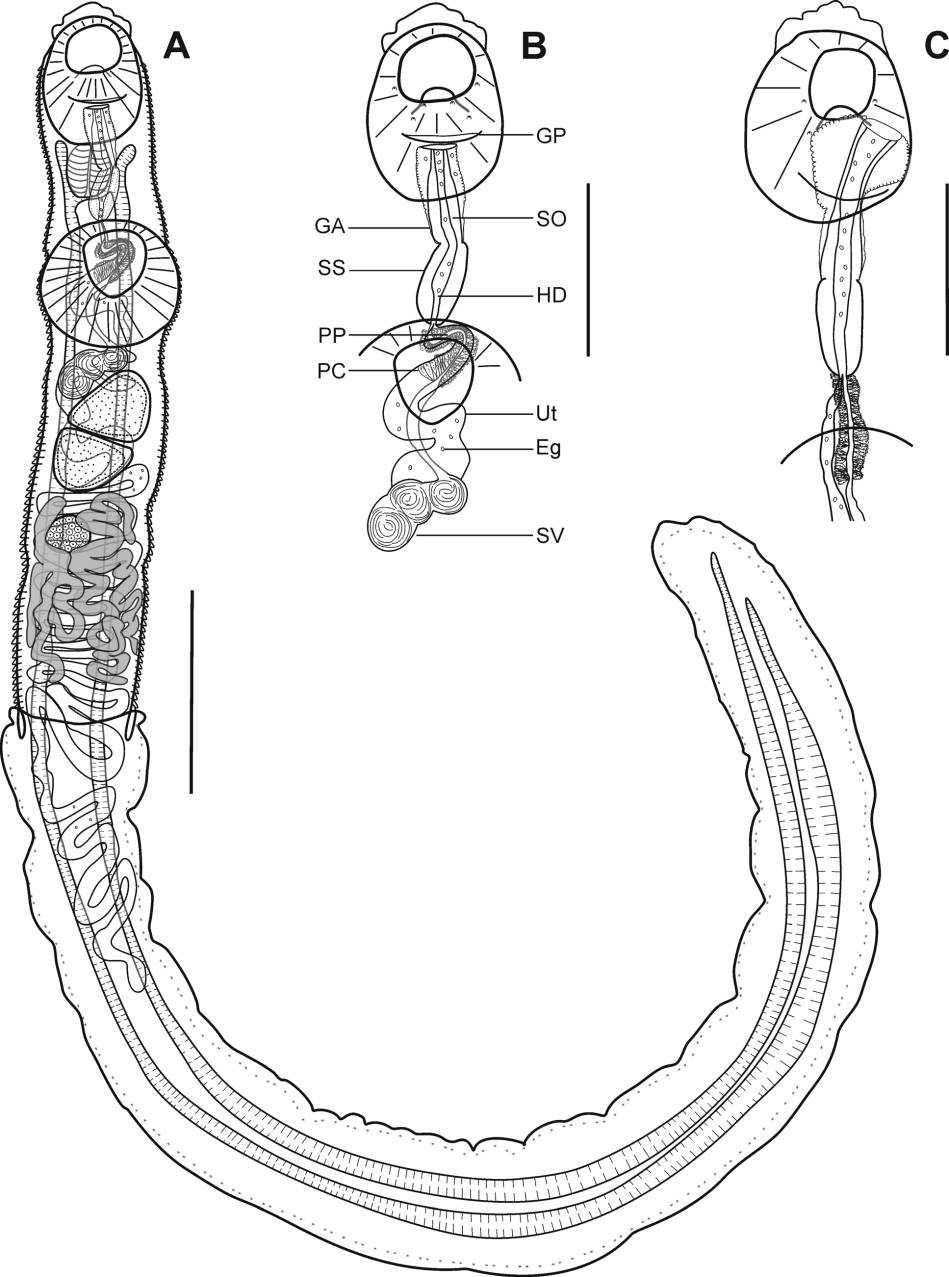
Adult of *Dinurus euthynni* ex *Auxis thazard*. (A) Complete specimen, ventral view, (B) detail of the terminal genitalia, ventral view, (C) detail of the terminal genitalia showing sinus-organ everted through the genital pore, ventral view. Scale-bars: A, 1000 μm; B, C, 600 μm. Abbreviations: Eg, eggs; GA, genital atrium; GP, genital pore; HD, hermaphroditic duct; PC, prostatic cells; PP, pars prostatica; SO, sinus organ; SS, sinus sac; SV, seminal vesicle; Ut, uterus.

Description (Figs. 1A–1C)

(Based on eight paragenophores and one hologenophore; measurements of paragenophores in Table 4 and hologenophore in description: Body elongate, narrow, rounded anteriorly and posteriorly, dorso-ventrally flattened, 3871 long. Maximum width at level of ventral sucker (*n* = 6), 971 or posterior body extremity (*n* = 3). Tegument covered with conspicuous plications. Forebody short, 1213, representing 31% of body length. Ecsoma well-developed, protruded, with irregular tegument, longer than body.

**Table 4:** Comparative metrical data of species from the family Hemiuridae found in the present study.

Species	*Dinurus euthynni* Yamaguti, 1934				*Lecithochirium floridense* Manter (1934)				*Lecithochirium synodi* Manter, 1931		
Source	Present study		Yamaguti [92]		Present study		Manter [58]	Bullard et al. [12]	Present study		Wang [90]
Locality	Atlantic Ocean, Rio de Janeiro, Brazil		Pacific Ocean, Japan		Atlantic Ocean, Rio de Janeiro, Brazil		Atlantic Ocean, Florida, USA	Atlantic Ocean, North Carolina, USA	Atlantic Ocean, Rio de Janeiro, Brazil		Pacific Ocean, Fujian, China
Host	*Auxis thazard*		*Katsuwonus pelamis*		*A*. *thazard*		*Paralichthys* sp.	*Pterois* cf. *volitans*	*A. thazard*		*Aluterus monoceros*
	*n* = 8		*n* = 2		*n* = 5		*n* = 17	*n* = 12	*n* = 7		*n* = 1
	Range	Mean		Range	Range	Mean	Range	Range	Range	Mean	
Body length	3526–5337	3977	–	–	1254–1637	1501	882–2242	547–1124	1678–2238	1993	2970
Body width	571–798	700	1230/1000	1000–1230	274–419	374	126–630	167–427	284–398	343	650
Ecsoma length	7029–10,747	8158	–/–	–	647–817 (*n* = 2)	732	92–1235	162 (*n* = 1)	446 (*n* = 1)	446	–
Total length	10,709–15,089	12,134	15,000/11,400	11,000–15,000	1174–2071 (*n* = 2)	1623	–	–	2334 (*n* = 1)	2334	–
Forebody length	837–1433	1075	–/–	–	303–376	314	176–504	138–314	270–413	383	–
Hindbody length	1625–2698	2093	–/–	–	698–1084	929	–	–	1103–1483	1314	–
Preoral lobe length	40–93	66	30/–	–	22–29	27	–	–	10–36	31	–
Oral sucker length	406–716	595	1000/790	800*–1000	91–104	99	75	57–99	104–136	118	125
Oral sucker width	405–609	530	950/680	–	90–110	102	129	48–102	97–153	116	136
Pharynx length	226–364	273	340/–	340	50–65	55	–	31–54	57–71	65	72
Pharynx width	181–286	229	–/–	–	56–69	62	–	31–52	45–69	61	68
Oesophagus length	–	–	–/–	–	15–37	29	–	–	9–12	10	–
Ventral sucker length	488–723	586	950/660	–	240–258	248	160	108–230	246–334	299	350
Ventral sucker width	531–730	637	1000/790	–	241–261	252	297	111–241	258–340	299	350
DIBAE	712–1026	833	–/–	–	153–186	166	–	–	185–212	191	–
Anterior testis length	237–376	313	–/290	290–450	64–118	98	–	31–111	135–187	161	224
Anterior testis width	271–419	330	–/300	450–623	64–100	91	–	37–128	90–154	125	200
Posterior testis length	276–365	322	–/450	–	82–143	112	–	–	150–191	172	208
Posterior testis width	285–388	335	–/450	–	88–97	92	–	–	97–183	144	260
Post-testicular region length	1072–2117	1518	–/–	–	552–856	782	–	–	613–1070	911	–
Seminal vesicle length	346–368 (*n* = 2)	357	–/–	–	111–184	159	–	–	163–211	179	250
Seminal vesicle width	144–155 (*n* = 2)	150	–/–	–	50–74	63	–	–	53–74	63	120
Sinus-sac length	256–408	326	–/–	–	95–120	105	68–141	85 (*n* = 1)	89–117	100	–
Sinus-sac width	118–219	165	–/–	–	59–87	76	–	–	42–66	57	–
Ovary length	159–269	213	340/260	260–340	91–107	101	–	43–140	98–129	106	160
Ovary width	186–267	244	490/–449	440–490	105–117	111	–	51–195	88–133	112	248
Vitellarium length	1017–1401	1202	–/–	–	104–130	123	–	–	135–216	177	–
Vitellarium width	388–587	475	–/–	–	106–180	149	–	–	104–230	176	–
Egg length	13–15 (*n* = 20)	14	16–18/–	16–18	12–15 (*n* = 20)	14	12–16	14–17 (*n* = 12)	14–17 (*n* = 20)	15	12–17
Egg width	9–12 (*n* = 20)	10	11/–	11	9–12 (*n* = 20)	10	9–10	7–10 (*n* = 12)	9–13 (*n* = 20)	10	8–11
Body length/body width	4.55–6.76	5.72	–	–	3.70–4.58	4.05	2.50–5.04	–	5.58–6.60	6.14	4.56
Oral/ventral sucker width	1:1.14–1.31	1:1.20	1:0.95/1:0.86	–	1:2.34–2.76	1:2.47	1:2–3	1:2.0–2.4	1:2.10–2.90	1:2.60	1:2.6
Ecsoma/body length, %	148–248	208	–/–	–	42–65 (*n* = 2)	54	20–33	15 (*n* = 1)	24 (*n* = 1)	–	–
Forebody/body length, %	23–31	27	–/–	–	18–24	21	25	26 –30	16–21	18	–
Post-testicular region/body length, %	30–43	38	–/–	–	44–56	52	–	–	32–50	45	–

*80 in the original description by Yamaguti [92], which we consider a misprint; *Abbreviation*: DIBAE, Distance of intestinal bifurcation from anterior extremity.

Pre-oral lobe distinct, 53 long. Oral sucker muscular, well developed, elongate oval (paragenophores) or transversely oval (hologenophore), 702 long, 765 wide. Prepharynx absent. Pharynx muscular, well developed, elongate-oval, 255 long, 159 wide. Oesophagus absent. Caeca blind, thick-walled with narrow lumen, shouldered at pharyngeal level, terminates close to posterior extremity of ecsoma. Ventral sucker muscular, well developed, subspherical (*n* = 2) or transversely oval (*n* = 7), 767 long, 812 wide, almost equal in size with oral sucker, 1:0.94, pre-equatorial.

Testes 2, obliquely tandem, contiguous, entire, pre-ovarian, median, in anterior half of hindbody, separated from ventral sucker; subspherical (*n* = 1) to subtriangular (*n* = 8), anterior testis, 339 long, 320 wide, posterior testis, 358 long, 301 wide. Post-testicular field 1253, representing 32% of body length. Seminal vesicle elongate, thin-walled, tripartite (quadripartite in one specimen), connected to pars prostatica by an aglandular duct, in anterior hindbody, immediately posterior to ventral sucker. Pars prostatica long, tubular, convoluted or straight (Figs. 1B and 1C), densely invested by gland-cells, anterodorsal to ventral sucker. Hermaphroditic duct straight within sinus sac. Sinus-sac elongate, muscular, 338 long, 178 wide. Permanent sinus-organ elongate, tubular, muscular, between mid-length of pharynx and genital pore; projecting into genital atrium and may evert outside through genital pore in some specimens (*n* = 3) (Fig. 1C). Genital atrium well developed. Genital pore median, posteroventral to oral sucker.

Ovary median or dextral (one paragenophore), entire, subspherical (*n* = 2) or transversely oval (*n* = 7), 199 long, 234 wide, in posterior half of hindbody, always separated from posterior testis by uterine coils, anterodorsal to vitellarium. Vitellarium seven elongate digitiform lobes (three sinistral and four dextral), between posterior testis and posterior body extremity, 843 long, 634 wide. Juel’s organ and Mehlis’ gland not observed. Uterus extensive in hindbody, extends up to one-fifth length of ecsoma. Metraterm not differentiated, terminal part of uterus joins male duct and passes into sinus-sac forming hermaphroditic duct. Eggs numerous, small, 13–16 × 07–10 (*n* = 10).

Excretory vesicle and excretory pore not observed.

*Remarks:* Specimens found in the present study correspond well to the generic diagnosis of *Dinurus* Looss, 1907 provided by Gibson [32] in having large and well developed ecsoma, plicated body surface, long pars prostatica densely invested by gland-cells and linked to the seminal vesicle by distinct aglandular duct, permanent sinus-organ, seminal vesicle constricted into portions and vitellarium seven digitiform tubes (three on one side, four on another).

Our specimens correspond in their morphology to *D. euthynni* described from the stomach of the skipjack tuna, *Katsuwonus pelamis* (*=Euthynnus pelamys*) (Linnaeus) in the Pacific Ocean by Yamaguti [92], particularly in the body shape, plicated tegument, long ecsoma (twice as long as the body), the presence of pre-oral lobe, and similar ratio of oral/ventral suckers (1:1.14–1.31 *vs* 1:0.95 in holotype *vs* 1:0.86 in paratype). However, they differ by having a narrower body (571–798 *vs* 1000–1230), longer pre-oral lobe (40–96 *vs* 30), smaller suckers (oral sucker 406–716 × 405–609 *vs* 1000 × 950 in holotype *vs* 790 × 680 in paratype; ventral sucker 480–723 × 531–730 *vs* 950 × 1000 in holotype *vs* 660 × 790 in paratype), and slightly shorter eggs (13–15 × 9–12 *vs* 16–18 × 11) (Table 4). Although there is variation in metrical data which is, in our opinion, related to differences in the fixation method (heat-killed fixation of our material *vs* fixation under the pressure of material in Yamaguti [92]), our specimens exhibit two key morphometric features consistent with *D. euthynni*, i.e., ratio of the body/ecsoma length and similar ratio of suckers.

Additionally, our specimens resemble specimens of *D*. *scombri* Yamaguti, 1934 more closely than any other congeners in possessing body of similar length (3526–5337 *vs* 4780 in holotype), similar oral/ventral sucker ratio (1:1.14–1.31 *vs* 1:1.06 in holotype), preoral lobe, tegument covered with conspicuous plications and vitellarium composed of seven elongate digitiform lobes. However, they differ from *D*. *scombri* in possessing ecsoma longer than body (1:1.49–2.48 *vs* 1:0.40 in holotype), longer and wider pharynx (226–364 × 181–286 *vs* 160–140 in holotype), longer and wider seminal vesicle (346–368 × 144–155 *vs* 190–74 in holotype) and longer extension of uterus within ecsoma (up to one-fifth length of ecsoma *vs* up to one-tenth in holotype).

*Dinurus euthynni* is a parasite of the stomach of scombrid fishes from the western Pacific Ocean. Since the original description, Mamaev [56] reported this species in *A*. *thazard* and *Euthynnus affinis* (Cantor) from the South China Sea, and Lester et al. [44] in the type host, *K. pelamis* from Helen Reef, Ponape, Papua New Guinea, Solomon Islands, Coral Sea, Fiji and Norfolk Island. This is the first record of *D*. *euthynni* in *A*. *thazard* off the Brazilian coast, southwestern Atlantic Ocean. Newly collected material of *D*. *euthynni* in the present study represents the fourth record of this species and provides the first detailed morphological description supplemented with DNA sequence data.

**Lecithochiriinae Lühe, 1901**

***Lecithochirium* Lühe, 1901**

### *Lecithochirium floridense* (Manter, 1934) Crowcroft, 1946

*Site of infection*: stomach.

*Infection rates*: 1 out of 3; 11 specimens in total.

*Representative DNA sequences*: OP458332 (28S); OP458339 (ITS2); OP418195–OP424998 (*cox*1).

*Voucher material*: 6 voucher specimens CHIOC–39760 a–f.

**Figure 2:**
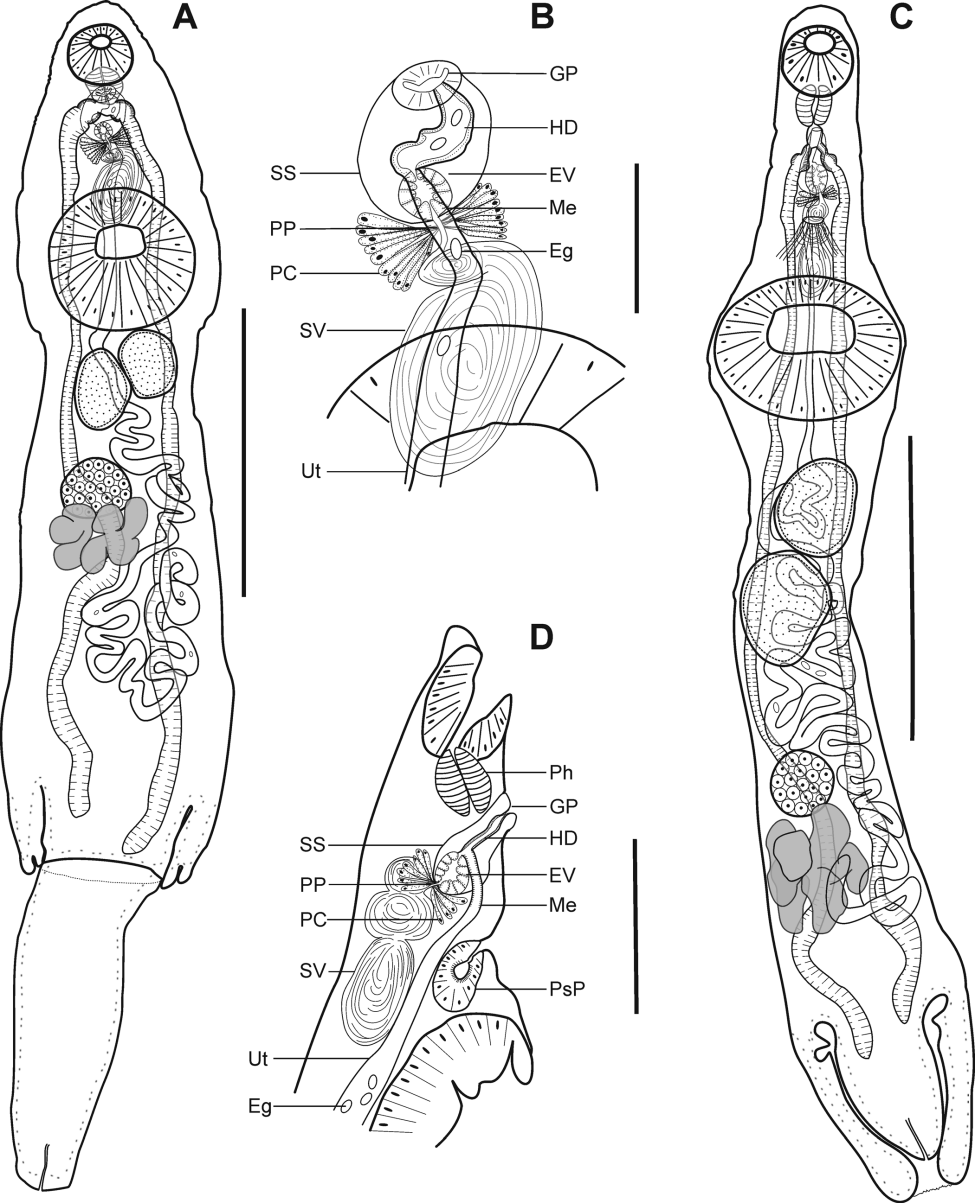
Adult of *Lecithochirium floridense* ex *Auxis thazard*. (A) Complete specimen, ventral view, (B) detail of the terminal genitalia, ventral view. Adult of *Lecithochirium synodi* ex *Auxis thazard*. (C) complete specimen, ventral view, (D) detail of the terminal genitalia, lateral view. Scale-bars: A, 600 μm; B, 100 μm; C, 500 μm; D, 200 μm. Abbreviations: Eg, eggs; EV, ejaculatory vesicle; GP, genital pore; HD, hermaphroditic duct; Me, metraterm; Ph, pharynx; PC, prostatic cells; PP, pars prostatica; PsP, presomatic pit; SO, sinus organ; SS, sinus sac; SV, seminal vesicle; Ut, uterus.

Description (Figs. 2A, 2B)

(Based on five paragenophores and one hologenophore; measurements of paragenophores in Table 4 and hologenophore in description): Body elongate, dorso-ventrally flattened, 1493 long. Maximum width close to posterior body extremity, 356. Tegument slightly rugose up to level of vitellarium. Forebody short 333, representing 22% of body length. Ecsoma well developed, withdrawn, protruded or partially extruded.

Pre-oral lobe distinct, 30 long. Oral sucker muscular, well developed, spherical, ventro-subterminal, 94 long, 103 wide. Prepharynx absent. Pharynx muscular, well developed, subspherical, 55 long, 57 wide. Oesophagus absent or short, 23 long. “Drüsenmagen” present. Presomatic pit absent. Caeca blind, with thick walls and narrow lumen, usually terminate in body or inside ecsoma when it is extruded (*n* = 2). Ventral sucker muscular, well developed, subspherical or elongate-oval 296 long, 241 wide, larger than oral sucker (1:2.3), pre-equatorial.

Testes 2, obliquely symmetrical, contiguous, entire, pre-ovarian, median, in anterior half of hindbody, contiguous with ventral sucker; dextral testis elongate oval, 117 long, 106 wide, sinistral testis subspherical 108 long, 110 wide. Post-testicular field 744, 50% of body length. Seminal vesicle thin walled, 133 long, 87 wide; bipartite, anterior portion subspherical, 24 long, 39 wide; posterior portion elongate-oval, larger than anterior, 109 long, 87 wide (Fig. 2B). Seminal vesicle between oral sucker and anterior half of ventral sucker, antero-dorsal to ventral sucker, connected to pars prostatica by an aglandular duct. Pars prostatica short, tubular, densely invested by gland-cells, anterior to ventral sucker (Fig. 2B). Ejaculatory vesicle conspicuous, spherical, enclosed within sinus-sac. Sinus-sac large, elongate-oval, anterior to intestinal bifurcation, with muscular wall, 95 long, 70 wide. Hermaphroditic duct enclosed within sinus-sac, curved, opens directly through the genital pore. Genital pore median, at level of pharynx.

Ovary dextral (*n* = 2) or sinistral (*n* = 4), entire, subspherical, 119 long, 122 wide, in anterior half of hindbody, always separated from posterior testis by uterine coils, anterodorsal to vitellarium. Vitellarium in 2 lateral compact masses, divided into three and four short lobes, 143 long, 232 wide, in mid-hindbody, contiguous with ovary. Juel’s organ and Mehlis’ gland not observed. Uterus coiled, restricted to body, or extending to ecsoma, up to its one third (*n* = 1) when it is extruded. Metraterm passes into sinus-sac ventrally, joins male duct just distally to ejaculatory vesicle forming hermaphroditic duct. Eggs numerous, small, 12–15 × 8–11 (*n* = 10).

Excretory vesicle not observed; excretory pore terminal.

*Remarks*: Specimens found in the present study correspond well to the generic diagnosis of *Lecithochirium* Lühe, 1901 provided by Gibson and Bray [31] and Gibson [32] in having well developed ecsoma, pre-oral lobe, tubular pars prostatica, vitellarium of two lateral masses divided into 3 and 4 short lobes, and eggs without polar filament.

Due to the morphological complexity of *Lecithochirium*, Bray [10] proposed a provisional key to species-groups. The species are classified into 24 groups based on modifications of the oral sucker, ratio of suckers, the presence or absence of presomatic pit, type of terminal genitalia, and shape of vitellarium. According to this classification, *L. floridense* belong to the “Musculus-group” based on the absence of the presomatic pit, the presence of the vitellarium of compact masses with distinct and short digitiform lobes, the terminal genitalia of the “musculus” type, the non-muscular seminal vesicle, and the absence of the internal elevations in ventral sucker.

Among the species belonging to the “Musculus-group”, our material most resembles *L. floridense* described by Manter [57] from the stomach of *Paralichthys* sp. (type host) and many other fish species in Tortugas, Florida, USA and later reported by Bullard et al. [12] from the stomach of the lionfish, *Pterois* cf. *volitans* in the Atlantic Ocean, off Beaufort, North Carolina, USA, particularly in possessing a preoral lobe, well developed ecsoma, genital pore at the level of the pharynx, bipartite seminal vesicle, suckers of similar ratio (1:2.34–2.76 *vs* 1:2.0–3.0 in Manter [58] *vs* 1:2.0–2.40 in Bullard et al. [12]), and eggs of similar size (12–15 × 9–12 *vs* 12–16 × 9–10 in Manter [58] *vs* 14–17 × 7–10 in Bullard et al. [12]). Our material differs from material of Manter [58] and Bullard et al. [12] in exhibiting higher minima for body size and size of most internal organs, and in lower maxima for body size, ecsoma and forebody length from specimens in material of Manter [58] (Table 4). Identification of the species based on morphological data was supported by the phylogenetic analyses (see below).

*Lecithochirium floridense* is a parasite of the stomach of a variety of marine fish species. After the original description, Manter [58] re-evaluated the material collected in Manter [58] and observed that there was more than one species among what he identified as *L*. *floridense*. Thereafter, he provided an updated list of hosts, which consisted of 21 species from 13 families. To date, *L*. *floridense* has been reported from fishes belonging to at least 16 families with the majority of records coming from the western Atlantic Ocean [12, 15, 48, 59, 65, 76, 81, 85, 88]. Parukhin [70] reported this species from *Haplobrotula gnathopus* (Regan) and *Scorpaena scrofa* Regan (=*Scorpaena natalensis*) collected off the South African coastline, i.e., Atlantic and Indian oceans. The only records of *L*. *floridense* from *Rhomboplites aurorubens* (Cuvier) (Lutjanidae) and *Pterois volitans* (Linnaeus) (Scorpaenidae) by Claxton et al. [15] and from *Syacium papillosum* (Linnaeus) (Cyclopsettidae) by Vidal-Martinez et al. [88] were supplemented with DNA sequence data, ITS1-5.8S-ITS2 and 28S rDNA, respectively. Our record of *A. thazard* infected with *L*. *floridense* off the Brazilian coast represents a new host and a new geographical record for this species.

### *Lecithochirium synodi* Manter, 1931

*Site of infection*: stomach.

*Infection parameters*: 1 out of 3; 12 specimens in total.

*Representative DNA sequences*: OP458330, OP458331 (28S); OP458337, OP458338 (ITS2); OP418194–OP424997 (*cox*1).

*Voucher material*: CHIOC–39761 a–f.

Description (Figs. 2C, 2D)

(Based on seven paragenophores and two hologenophores; measurements of paragenophores in Table 4 and hologenophores in description: Body elongate, narrow, dorso-ventrally flattened. Maximum width at ventral sucker level, 319–392. Tegument slightly rugose up to level of posterior testis. Forebody short, 420–441. Ecsoma well developed, withdrawn or protruded.

Pre-oral lobe distinct, 16–26 long. Oral sucker muscular, well developed, spherical, ventro-subterminal, 142–143 long, 129–134 wide. Prepharynx absent. Pharynx muscular, well developed, subspherical, 64–69 long, 66 wide. Oesophagus absent or short. “Drüsenmagen” present. Presomatic pit glandular, between genital pore and anterior margin of ventral sucker (Fig. 2D). Caeca blind, with thick walls and narrow lumen, usually terminate in body or inside ecsoma when everted (*n* = 1). Ventral sucker muscular, well developed, subspherical or transversely oval, 346–368 long, 280–359 wide, larger than oral sucker (1:2.1–2.8).

Testes 2, obliquely tandem, contiguous, entire, pre-ovarian, median, in anterior half of hindbody, separated from ventral sucker; anterior testis elongate oval or spherical, 133–195 long, 133–170 wide, posterior testis elongate-oval, 161–221 long, 128–182 wide. Seminal vesicle thin walled, 150–167 long, 47–56 wide; tripartite, anterior portion subspherical, 42–51 long, 53–62 wide; middle portion transversely oval, 33–41 long, 43–49 wide; posterior portion elongate oval, 62–88 long, 47–48 wide (Fig. 2D). Seminal vesicle antero-dorsal to ventral sucker, connected to pars prostatica by aglandular duct. Pars prostatica short, tubular, densely invested by gland-cells (Fig. 2D). Ejaculatory vesicle conspicuous, spherical, enclosed within sinus-sac. Sinus-sac large, pyriform, between pharynx and presomatic pit, with muscular wall, 83–90 long, 48–63 wide. Hermaphroditic duct enclosed within sinus-sac, straight, opens directly through the genital pore. Genital pore median, just posterior to pharynx.

Ovary dextral (*n* = 5) or sinistral (*n* = 4), subspherical or transversely oval, entire, 87–118 long, 104–141 wide, in posterior half of hindbody, always separated from posterior testis by uterine coils, adjacent or contiguous with vitellarium. Vitellarium in 2 lateral compact masses, divided into three and four digitiform lobes, 299–233 long, 203–320 wide, in posterior hindbody, contiguous with ovary. Juel’s organ and Mehlis’ gland not observed. Uterus coiled, restricted to body. Metraterm passes into sinus-sac ventrally, joins male duct just distally to ejaculatory vesicle forming hermaphroditic duct. Eggs numerous, small, oblong 13–16 × 9–11 (*n* = 10).

Excretory vesicle not observed; excretory pore terminal.

*Remarks*: Specimens found in the present study correspond well to the generic diagnosis of *Lecithochirium* in characters as mentioned above. Following the key to species-groups of *Lecithochirium* proposed by Bray [10], our specimens belong to the “Synodi-group” based on the presence of the glandular presomatic pit, vitellarium of compact masses with distinct and short digitiform lobes, the terminal genitalia of the “musculus” type, the non-muscular seminal vesicle, and the absence of the internal elevations in ventral sucker.

In comparison with species from the “Synodi-group”, our specimens can be distinguished from *L*. *exodium* McFarlane, 1936, *L*. *canadus* Bilqees, 1972*, L. harpodoni* Bilqees, 1972 and *L*. *leiperi* Gupta & Singh, 1981 based on the position of testes (oblique *vs* symmetrical); from *L*. *acutum* Chauhan, 1945 in the shape of pre-oral lobe (dome *vs* nipple); from *L*. *sinaloense* Bravo-Hollis, 1956 in the position of testes (contiguous *vs* never contiguous, separated by uterine coils); from *L*. *texanum* (Chandler, 1941), *L*. *spindale* Bilqees, 1972, *L. perfidum* Gomes, Fabio & Rolas, 1972 and *L*. *musculoatrium* Bilqees, 1972 in possessing a smaller sucker ratio (1:2.10–2.90 *vs* 1:>3 *vs* 1:4.1–4. *vs* 1:3.18–3.5 *vs* 1:3, respectively); from *L*. *taboganus* (Sogandares-Bernal, 1959) in possessing a larger sucker ratio (1:2.10–2.90 *vs* 1:1.68–1.95, respectively); from *L*. *kawalea* Yamaguti, 1970 in possessing shorter oesophagus (9–12 *vs* 80); and from *L*. *polynemi* Chauhan, 1945 in position of seminal vesicle (reaching the anterior margin of the ventral sucker *vs* entirely anterior to the ventral sucker).

Our specimens are morphologically similar to *L*. *bothi* Yamaguti, 1970, *L*. *kawakawa* Yamaguti of Bray et al. [7] and *L*. *synodi* Manter, 1931 in possessing oblique testes and similar ratio of suckers (1:2.10–2.90 *vs* 1:2.0–2.4 *vs* 1:2.16–3.52 *vs* 1: >2, respectively). However, they can be distinguished from *L*. *bothi* in possessing shorter oesophagus (9–12 *vs* 40–110), smaller and narrow seminal vesicle (163–211 × 53–74 *vs* 200–450 × 90–170) and slightly shorter eggs (14–17 × 9–13 *vs* 16–21 × 9–12). Although our specimens closely resemble specimens of *L*. *kawakawa* collected from *E*. *affinis* in the Great Barrier Reef by Bray et al. [7] in possessing the genital pore posterior to the pharynx, tripartite seminal vesicle, pyriform sinus-sac and testes separated from ventral sucker, they differ by having a shorter distance between testes and ovary (106–233 *vs* 465–555). Furthermore, our specimens differ from the original description of *L*. *kawakawa* by Yamaguti [93] in possessing shorter oesophagus (9–12 *vs* 30–150), smaller seminal vesicle (163–211 × 53–74 *vs* 180–420 × 50–150), in position of genital pore (just posterior to pharynx *vs* at pharynx level), slightly smaller eggs (14–17 × 9–13 *vs* 16–26 × 9–14) and in shape of sinus-sac (pyriform *vs* subspherical). In comparison with specimens of *L*. *kawakawa* of Bray [10], our specimens differ in partition of seminal vesicle (tripartite *vs* bipartite) and in shape of sinus-sac (pyriform *vs* transversely oval).

Morphologically, our material most closely resembles *L. synodi* described from the lizard fish *Synodus foetens* (Linnaeus) (type host) and the summer flounder *Paralichthyis dentatus* (Linnaeus) collected off Beaufort, North Carolina and Tortugas, Florida, USA [57, 59] particularly in possessing a pre-oral lobe, conspicuous and glandular pre-somatic pit, tripartite seminal vesicle, pyriform sinus-sac, genital pore posterior to pharynx, and similar oral/ventral sucker ratio (1:2.10–2.90 *vs* 1:2.2–2.8 in Manter [59]). However, our specimens differ from material of Manter [57] in narrower body (284–398 *vs* 500–800), lower maxima of body length (2238 *vs* 4800) and slightly larger eggs (14–17 × 9–13 *vs* 12–16 × 7–9). Our material differs from specimens collected from the unicorn leatherjacket filefish *Aluterus monoceros* (Linnaeus) in the South China Sea and identified by Wang [90] as *L. synodi* in smaller and narrower body (1678–2238 × 284–398 *vs* 2970 × 650], smaller testes (anterior testis 135–187 × 90–154; posterior testis 150–191 × 97–183 *vs* anterior testis 224 × 200; posterior testis 208–260), smaller and narrow seminal vesicle (163–211 × 53–74 *vs* 250 × 120) and smaller ovary (98–129 × 88–133 *vs* 160 × 248) (see Table 4).

Although limited with sequence data, we followed a model of trematode species recognition proposed by Bray et al. [8] for the identification of our specimens as *L*. *synodi* based on their morphology (see above) and geographical distribution of their hosts. *Lecithochirium bothi* and *L*. *kawakawa* have thus far been reported to have their geographical distributions in the Indian and Pacific oceans [7, 10, 90, 94], whereas *L. synodi* has been reported in the north-western Atlantic Ocean [37, 57, 59]. Although *L*. *synodi* has been reported from the unicorn leatherjacket filefish *Al. monoceros* in the South China Sea, north-western Pacific Ocean by Wang [90], this record should be interpreted with caution. The specimens of Wang [90] are morphologically similar to other two species, *L*. *bothi* and *L*. *kawakawa* reported in the Pacific Ocean. Previous reports on fish host spectrum of the three species suggest that all have low host specificity. *Lecithochirium bothi* was reported in bothiid and mullid fishes, *L*. *kawakawa* in platycephalid, scombrid and sparid fishes, and *L*. *synodi* in monacanthid, paralichthyid and synodontid fishes. In our study, *L*. *synodi* was for the first time reported from scombrid fish *A*. *thazard* and this is the first record of *L*. *synodi* off the Brazilian coast, south-western Atlantic Ocean. Our newly collected material of *L*. *synodi* has allowed us to provide detailed morphological description of the species and generate DNA sequence data.

**Figure 3:**
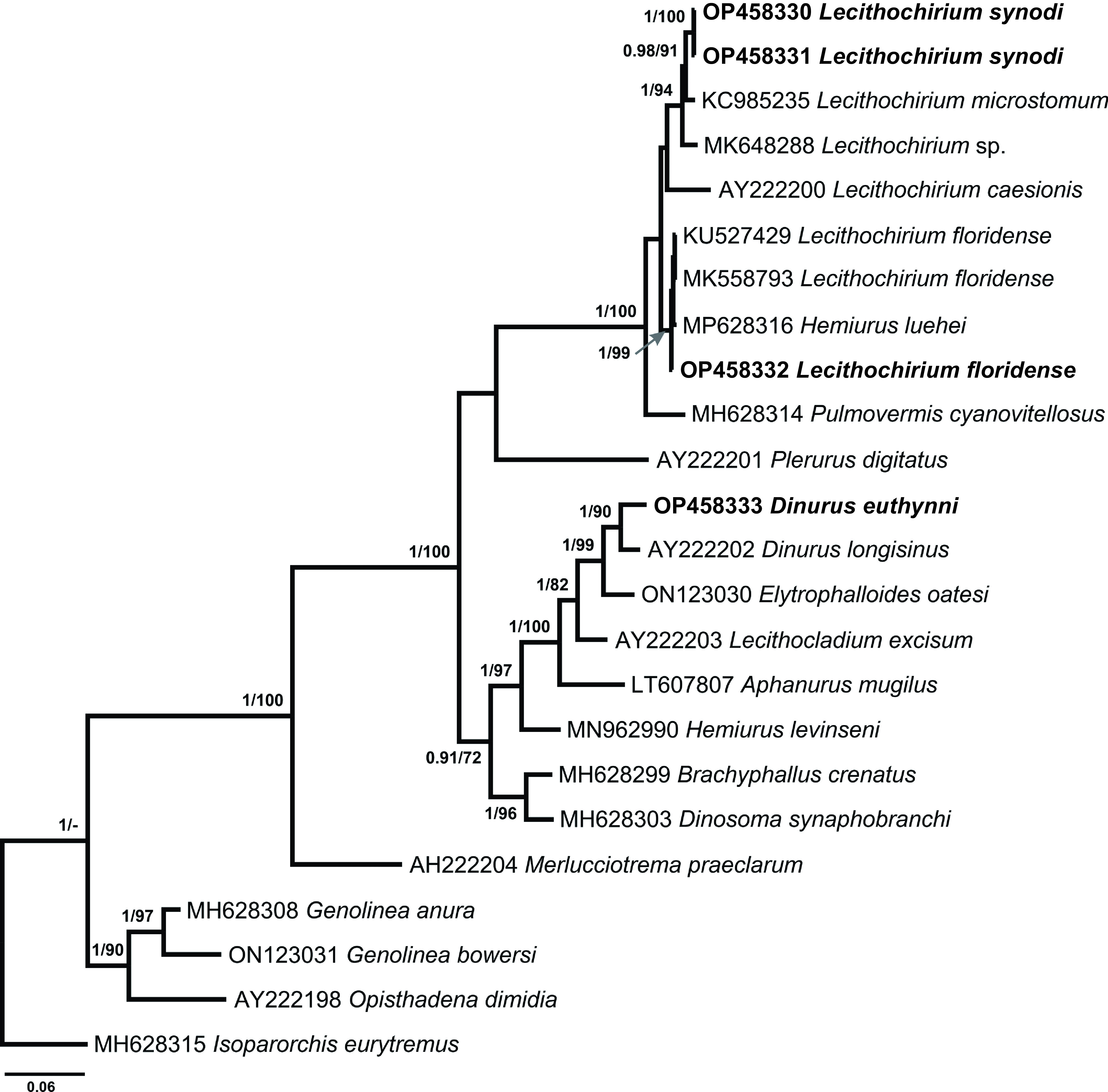
Phylogram from Bayesian inference (BI) analysis based on the 28S rDNA sequences of the Hemiuridae. Nodal support values are given as BI/ML (maximum likelihood). Support values lower than 0.90 (BI) and 70 (ML) are not shown. The scale-bar indicates the expected number of substitutions per site. Newly generated sequences are highlighted in bold.

*Molecular results*: Figure 3 represents the phylogram obtained from BI analyses based on Alignment 4. Novel sequences of three species were positioned in two clades with the members of the family Hemiuridae. Consequently, the taxonomic positions of *D*. *euthynni* within the genus *Dinurus*, *L*. *floridense* and *L*. *synodi* within the genus *Lecithochirium* were confirmed by the phylogenetic analyses.

Our sequence of *D*. *euthynni* (OP458333) clustered with the sequence of *D*. *longisinus* (AY222202) collected from *Coryphaena hippurus* (Linnaeus) in Jamaica; the sequence divergence was 2.9% (32 nt). The two sequences of *L*. *synodi* (OP458330; OP458331) were identical and clustered with *L*. *microstomum* Chandler, 1935 (KC985235) collected from *Trichiurus lepturus* Linnaeus in USA and unidentified species of *Lecithochirium* (MK648288) from *T. lepturus* in Mexico in a strongly supported subclade. The sequence of *L*. *floridense* (OP458332) clustered with sequences of two isolates of the same species found in *S*. *papillosum* from Yucatan Shelf, Mexico (MK558793) and in *P*. *volitans* from Northern Gulf of Mexico, USA (KU527429), and a sequence identified as *Hemiurus luehei* Odhner, 1905 (MH628316) found in *Ophidion rochei* Müller from the Black Sea, Ukraine. The intraspecific divergence between four isolates was 0–0.2% (0–2 nt). The interspecific divergence within the clade of *Lecithochirium* spp. was 1.2–5.2% (13–55 nt) with *L*. *synodi* and *L*. *microstomum* exhibiting the lowest interspecific divergence and *L*. *caesionis* and unidentified *Lecithochirium* sp. (MK648288) exhibiting the highest interspecific divergence. The ITS2 sequence of *L*. *floridense* (OP458339) differed from sequences of the same species (KU527428 and KU527429) by 2.2% (11 nt). Both *cox*1 regions sequenced for *Lecithochirium* spp. in our study demonstrated high interspecific divergence – 19.71% (97 nt) and 20.67% (202 nt).

## Discussion

Despite its wide distribution in the Atlantic Ocean and its economic value for regional commercial fisheries, *A. thazard* has not been a frequent target of fish parasitological investigations in Brazil. The present study is the first to apply both morphological and molecular techniques to explore the diversity of digenean trematodes of the frigate tuna *A*. *thazard* collected off the coast of Rio de Janeiro, Brazil. Despite a small sample size of *A. thazard* in our study, we recorded six species of digenean trematodes, namely: the bucephalid *Rhipidocotyle* cf. *angusticolle*, the didymozoid *Didymocystis* sp. 6 *sensu* Louvard et al. [49], the fellodistomid *Tergestia* sp*.,* and three hemiurids *D. euthynni, L. floridense* and *L. synodi*. For three of these species – *R*. cf. *angusticolle*, *L*. *floridense* and *L*. *synodi* – the frigate tuna was reported as a new host and four – *Didymocystis* sp. 6 *sensu* Louvard et al. [49], *D. euthynni*, *L*. *floridense* and *L*. *synodi* – were reported in Brazil for the first time. With our new records, the number of digenean trematodes of *A. thazard* in Brazil increased from two to eight species, with hemiuroid trematodes being most diverse in this fish.

Specimens putatively identified in our study as *R. angusticolle* were found for the first time in the frigate tuna. *Rhipidocotyle angusticolle* is a stenoxenous parasite and has previously been reported only in scombrid fishes in the western Atlantic Ocean [14, 17, 68]. With 64 species parasitizing freshwater and marine fishes worldwide [68] the genus *Rhipidocotyle* is represented by only four species in marine fishes in Brazil, namely: *R*. *angusticolle*, *R. fluminensis* Vicente & Santos, 1973, *R. pentagonum* (Ozaki, 1924), and *R. quadriculatum* Kohn, 1961. They are all parasites of scombrid fishes in the region [21].

The first records of two hemiuroids *D*. *euthynni* and *Didymocystis* sp. 6 *sensu* Louvard et al. [49] in the Atlantic Ocean demonstrate that the geographical distribution of these species is wider than formally known. Previous records were restricted to the Pacific Ocean [44, 49, 56, 92]. The wide distribution of these species is most likely associated with the distribution of the frigate tuna. Mamaev [56] reported *D*. *euthynni* from *A. thazard* in the South China Sea and Louvard et al. [49] reported *Didymocystis* sp. 6 from *A. thazard* in Moreton Bay, Australia. The identification of *Didymocystis* sp. 6 *sensu* Louvard et al. [49] was based on DNA sequence comparison, while DNA sequence data for *D*. *euthynni* were not available for comparison. Thus, based on DNA sequence data, *Didymocystis* sp. 6 *sensu* Louvard et al. [49] was confirmed to be an oioxenous parasite infecting *A. thazard*, whereas *D. euthynni* is known as a stenoxenous parasite infecting fishes from the family Scombridae.

*Didymocystis* is a large genus represented by over 30 species ubiquitously distributed in marine ecosystems and predominantly parasitizing scombrid fishes [42, 49, 80]. In Brazil, eight species of *Didymocystis*, namely: *Di*. *bifasciata* (Yamaguti, 1970), *Di*. *dissimilis* Yamaguti, 1938, *Di*. *kamegaii* (Yamaguti, 1970), *Di*. *lamotheargumedoi* Kohn & Justo, 2008, *Di*. *neothunni* (Yamaguti, 1970), *Di*. *pinnicola* (Yamaguti, 1970), *Di*. *scomberomori* (MacCallum & MacCallum, 1916) and *Di*. *wedli* Ariola, 1902 have previously been reported from scombrids collected in the same region as the present study.

The genus *Dinurus* currently accommodates 13 nominal species widely distributed in freshwater and marine ecosystems and parasitizing fishes from at least seven families (Alestidae, Chirocentridae, Clupeidae, Coryphaenidae, Engraulidae, Scombridae, and Stromateidae) [4, 26, 33, 35, 78, 92]. Our record of *D*. *euthynni* off the Brazilian coast is the first report of this species in the Atlantic Ocean and it increases the diversity of *Dinurus* in Brazil from three to four species. Three species of *Dinurus* – *D*. *barbatus* (Cohn, 1902) Looss, 1907, *D. tornatus* Rudolphi, 1819, and *D*. *scombri* Yamaguti, 1934 – were previously found parasitizing coryphaenid and scombrid fishes [1, 41, 47].

Species of *Lecithochirium* found in our study – *L. floridense* and *L*. *synodi* – demonstrated exceptionally low host specificity by infecting fishes of different species, families, and orders. *Lecithochirium floridense* is known from a high variety of marine fishes [12], however, only the records from *Rh. aurorubens* (Lutjanidae), *P. volitans* (Scorpaenidae), and *S. papillosum* (Cyclopsettidae) have been confirmed based on DNA sequence data. Our DNA based record of *L. floridense* in *A. thazard* (Scombridae) has again confirmed its euryxenous nature*. Lecithochirium synodi* is currently known to parasitise *Sy. foetens* (Synodontidae), *Pa. dentatus* (Paralichthyidae), *Al. monoceros* (Monacanthidae), and *A. thazard* (Scombridae) with only the latter record rooted in DNA sequence data. Based on the studies to date, species of *Lecithochirium* exhibit the full range of host specificities [10, 13, 21, 54]. In addition to *L. floridense* and *L*. *synodi*, several other species of the genus, namely: *L. bothi, L. genypteri, L. kawaka, L. musculus*, *L. microstomum*, *L. monticellii*, *L. rufoviride*, and *L. furcolabiatum*, have been demonstrated to infect a variety of fishes from more than one family [10, 13, 21, 27, 31, 90].

Seven species of *Lecithochirium* have previously been reported in Brazil, namely: *L*. *monticellii* (Linton, 1898), *L*. *imocavum* (Looss, 1907), *L*. *microstomum* Chandler, 1935, *L*. *texanum* (Chandler, 1941), *L*. *zeloticum* (Travassos, Teixeira de Freitas & Buhrnheim, 1966), *L*. *manteri* Teixeira de Freitas & Gomes, 1971 and *L*. *perfidum* Gomes, Fabio & Rolas, 1972 [6, 21]. Among nine Brazilian species of *Lecithochirium*, four are oioxenous, one is stenoxenous, and four are euryxenous.

*Lecithochirium* is one of the most species-rich genera within the Hemiuridae, with over 100 species parasitizing marine fishes from several orders [54]. The taxonomy of the genus remains in a controversial state due to the presence of morphologically similar species, poor morphological descriptions for the majority of species, and the lack of DNA sequences which hinders elucidation of its composition based on phylogenetic analysis [10, 31, 54, 60]. There is an obvious need for a thorough revision of the genus based on additional datasets that combine information on species morphology, DNA sequences and host distribution as proposed by Bray et al. [8]. It is worth noting that the findings of the current study do not support the previous study of Sokolov et al. [84] that published the DNA sequence of *He. luehei*. The results of our phylogenetic and comparative sequence analyses demonstrated that the sequence of the isolate identified as *He. luehei* clustered among the isolates of *L. floridense*. Thus, our results indicate incorrect identification of the *He. luehei* isolate. Sequences of *L. floridense* were not included in the analysis of Sokolov et al. [84] and, consequently, the phylogenetic position of the genus *Hemiurus* being closely related to the genus *Lecithochirium* within the Hemiuridae was erroneous.

Digenean trematodes represent the most diverse group of parasites in *A. thazard*. Previous studies performed by Mamaev [56] and Yamaguti [94] showed high species richness of digeneans in *A. thazard* in the Pacific Ocean, with 12 and 8 species being found, respectively (see Table 1). Recently, however, Louvard et al. [49] found eight didymozoids in the frigate tuna from Moreton Bay, Australia. The fauna of digeneans of *A. thazard* in the Atlantic Ocean*,* including the results of our study, currently accounts for ten species. Prior to our study, *R. pentagonum* was the only species recorded in the frigate tuna in both the Atlantic and Indo-Pacific regions. In our study, *D. euthynni* and *Didymocystis* sp. 6 *sensu* Louvard et al. [49] known from the frigate tuna in the South China Sea [56] and in Moreton Bay, Australia were discovered in the frigate tuna in the Atlantic Ocean for the first time.

To date, 63 nominal species of digenean trematodes from 10 families, including our data, have been reported in scombrid fishes in Brazil [21, 38]. The skipjack tuna *K. pelamis* and blackfin tuna *Th. atlanticus* (Lesson) are the scombrid hosts with the highest diversity of digeneans in the region, each known as the host for at least 15 digenean species [21, 38]. Of the 63 species of digeneans, there are only three species – two bucephalids, *R*. *angusticolle* and *R. pentagonum*, and one didymozoid *M. kawakawa* – that *A. thazard* shares with the other species of scombrid fishes in the region, namely: *E. alletteratus*, *K. pelamis*, *Th. atlanticus*, and *Scomber colias*. We believe that this information is likely to change when the diversity of digeneans from *A. thazard* becomes better known in Brazil.

The present study brings to light new information on the digenean diversity of the frigate tuna in Brazil and presents novel sequence data and data on host association and geographical distribution of six digenean species. Further large-scale investigations including seasonal monitoring and the application of an integrative taxonomic approach will uncover the true species diversity of digenean trematodes in *A. thazard* from the Atlantic Ocean and beyond.
